# Moya moya: étiologie rare d’accident vasculaire cérébral ischémique chez l’enfant: à propos d’un cas

**DOI:** 10.11604/pamj.2017.28.192.8740

**Published:** 2017-11-01

**Authors:** Radia Chibli, Youssef Omor, Nadir Slimani Sebbouba, Moulay Rachid El Hassani, Mohamed Jiddane, Meriem Fikri

**Affiliations:** 1 Service de Neuroradiologie, Hôpital des Spécialités, Rabat, Maroc

**Keywords:** Moya, vascularite, AVC, angioscanner, angio-IRM, angiographie, Moya, vasculitis, stroke, angioscanner, angio-MRI, angiography

## Abstract

La maladie de Moya Moya est une maladie angiogénique, caractérisée par un rétrécissement de l'artère carotide interne distale qui s'étend aux segments proximaux des artères cérébrales moyennes et antérieures, induisant la formation de vaisseaux de suppléance. Ces derniers proviennent des collatérales parenchymateuses, perforantes, leptoméningées et autres anastomoses transdurales. Ces vaisseaux collatéraux ont un aspect caractéristique à l'angiographie formant un nuage de fumée : réseau Moya Moya. Son étiologie reste encore mal élucidée et représente 10 à 15% des causes d'accidents vasculaires cérébraux (AVC), avec 2 pics d'âge où l'atteinte est plus fréquente: les enfants autour de 5 ans et les adultes autour de 40 ans. Son évolution peut être lente avec des symptômes intermittents ou être fulminante avec un déclin neurologique rapide. Les données actuelles montrent l'importance du traitement chirurgical comme méthode de référence pour la prise en charge du syndrome de Moya en particulier chez les patients avec des symptômes progressifs et récidivants.

## Abstract (en)

Moya moya disease is an angiogenic disease characterized by the narrowing of the distal internal carotid artery extending to the proximal segments of the middle and anterior cerebral arteries, inducing collateral vessels formation. These vessels come from the collateral parenchymal vessels, the perforating vessels, leptomeningeal vessels and other transdural anastomoses. These collateral vessels have a characteristic appearance on angiography, forming a cloud of smoke: net-like moyamoya. Its etiology is still poorly understood. Moyamoya disease accounts for 10-15% of the causes of stroke, with 2 age peaks at which its occurrence is more frequent: children around 5 years old and adults around 40 years old. Its evolution can be slow with intermittent symptoms or fulminant with fast neurological deterioration. The current data show the role of surgery as the gold standard for the treatment of moyamoya syndrome, in particular in patients with progressive and recurrent symptoms.

## Introduction

La maladie de Moya Moya est une maladie angiogénique, caractérisée par un rétrécissement de l'artère carotide interne distale qui s'étend aux segments proximaux des artères cérébrales moyennes et antérieures. Décrite pour la première fois en 1957 au Japon par Takeuchi et Shimizu. Elle est caractérisée par le développement de ces occlusions induisant la formation de vaisseaux de suppléance. Ces derniers proviennent des collatérales parenchymateuses, perforantes, leptoméningées et autres anastomoses transdurales. Ces vaisseaux collatéraux ont un aspect caractéristique à l'angiographie formant un nuage de fumée : réseau Moya Moya. Son étiologie reste encore mal élucidée et représente 10 à 15% des causes d'accidents vasculaires cérébraux (AVC).

## Patient et observation

**Figure 1:**
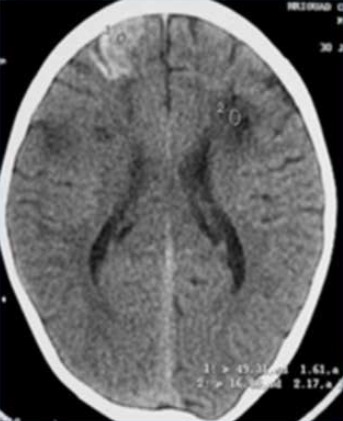
TDM cérébrale C: plage lésionnel hypodense frontale gauche avec attraction de la corne frontale homolatérale. Il s’y associe un autre foyer lésionnel frontal droit

**Figure 2:**
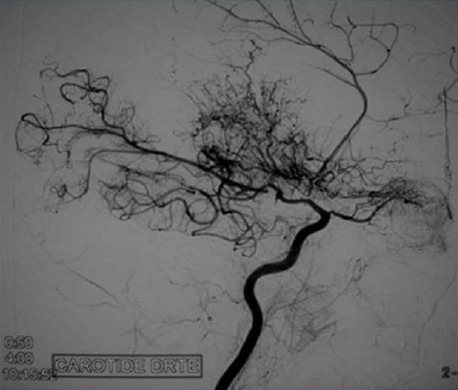
Artériographie cérébrale avec injection sélective de la carotide interne droite: montrant son interruption au niveau de son segment supraclinoidien,visibilité du développement d’un réseau Moya qui permet l’opacification de la cérébrale antérieure et à moindre degré la sylvienne

**Figure 3:**
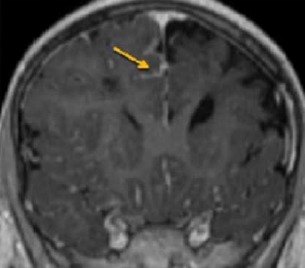
IRM en coupe coronale T1 injectée montrant un rehaussement leptomeningé (flèche): « Ivy sign »

Il s'agit d'un enfant de sexe masculin âgé de 4 ans, ayant comme antécédent un déficit régressif de l'hémicorps droit un an auparavant, et qui a présenté un tableau d'AVCI avec un déficit partiel de l'hémicorps gauche installé il y' a 15 jours. Le patient a bénéficié d'un scanner cérébral ainsi que d'une artériographie. Le scanner cérébral en contraste spontané montre un foyer lésionnel hypodense frontal gauche avec élargissement des sillons corticaux en regard et attraction des la corne frontale du ventricule latéral homolatéral en rapport avec un foyer ischémique séquellaire frontal droit avec hyperdensités corticales (Figure 1). D'autres petites lésions ischémiques sont notées au niveau de la substance blanche du centre oval droit. Devant ce tableau d'accidents vasculaires cérébraux ischémiques d'âge différents chez l'enfant, de nombreuses étiologies ont été recherchées notamment cardiaques et métaboliques. Une angiographie cérébrale a été réalisée dans le cadre du bilan étiologique, par ponction de l'artère fémorale, et cathétérisme sélectif de l'artère carotide interne droite. Elle a montré l'interruption de cette carotide au niveau de son segment supraclinoidien ainsi que le développement d'un réseau Moya formé de fines anastomoses provenant de perforantes ainsi que de l'artère ophtalmique qui permet l'opacification de l'artère cérébrale antérieure et à moindre degré la sylvienne (Figure 2). Visualisation de l'« Ivy sign » en IRM (Figure 3).

## Discussion

L'incidence des AVC ischémiques chez l'enfant est évaluée entre1,2 et 2,7/100000 par an, dont 10 à 15% représentées par le Moya Moya [1], qui est caractérisée par: l'occlusion progressive des portions distales des artères carotides internes supraclinoidiennes, et/ou des portions proximales des artères cérébrales moyennes et/ou antérieures, par le développement d'un réseau vasculaire irrégulier de suppléance réalisant l'aspect en « nuage de fumée », Moya Moya en japonais. Ces vaisseaux sont pathologiques, fragiles, pouvant présenter des dilatations et des microanévrysmes d'où le risque de rupture et d'hémorragie intracérébrale et/ou ventriculaire. Le terme de Moya Moya se réfère uniquement aux résultats distinctifs sur l'artériographie cérébrale indépendamment de la cause. Par définition: on distingue La maladie de Moya Moya qui est idiopathique et isolée, du syndrome de Moya Moya qui lui est associée à d'autre maladies telles que : la neurofibromatose type 1, artériosclérose, trisomie 21, néoplasie, traumatisme crânien, méningite, radiothérapie antérieure, drépanocytose et maladie auto-immune. Dans la maladie de Moya Moya les résultats artériographiques sont pathognomoniques. L'atteinte est bilatérale, même si la gravité peut varier d'un coté à l'autre. Cependant 40% des patients présentent initialement une atteinte unilatérale pouvant progresser et aboutir à une atteinte bilatérale.

Sur le plan épidémiologique, le syndrome de Moya-Moya a été décrit pour la première au Japon. Il y a 2 pics d'âge où l'atteinte est plus fréquente: les enfants autour de 5 ans et les adultes autour de 40 ans. Il existe une prédominance féminine : les femmes étant 2 fois plus touchées que les hommes. Le Moya Moya est la plus fréquente maladie cérébro-vasculaire pédiatrique au Japon avec une prévalence d'environ 3/100 000 [1]. Bien que la cause et la pathogenèse de la maladie de Moya Moya soient encore mal élucidées, les facteurs génétiques jouent un rôle majeur: l'incidence familiale de parents atteints au premier degré au Japon est de 10%, et 6% dans une récente série aux États-Unis [1]. Ainsi la pathogenèse du Moya-Moya implique très probablement des facteurs aussi bien génétiques qu'environnementaux. La physiopathologie est mal connue. Elle serait liée à une artériopathie chronique idiopathique, avec épaississement progressif de la paroi des artères cérébrales. ce qui entraine la production de facteurs angiogéniques responsables de la formation de néovaisseaux de suppléance, qui sont de trois types: 1) vaisseaux des Moya Moya: formés via les artères perforantes intraparenchymateuses; 2) vaisseaux collatéraux leptoméningés de l'artère cérébrale postérieure: donne l'« Ivy sign » en IRM (Figure 3) et les vaisseaux collatéraux transduraux de l'artère méningée moyenne, l'artère temporale superficielle, l'artère ethmoïdale et/ou de l'artère occipitale. Les patients atteints de Moya Moya se présentent avec des signes et des symptômes résultants des changements dans la circulation de l'artère carotide interne, qui peuvent être classés en deux groupes: lésion ischémique produisant des AIT ou AVCI ou des conséquences de phénomènes compensatoires à cette ischémie: hémorragie par fragilité vasculaire, anévrysme, dilatation transdurale unilatérale [2]. La variation du degré d'atteinte artérielle, la vitesse de progression, les zones corticales atteintes et la réponse à la réduction de l'apport sanguin contribuent à expliquer la diversité des signes cliniques et des présentations, à type de céphalées, AIT, AVC ischémiques, AVC hémorragiques par rupture d'anévrysme du système vertébro-basilaire, hémorragies sous arachnoïdiennes surtout chez l'adulte ou de convulsions, épilepsie, détérioration intellectuelle, et retard scolaire [2]. Sur le plan imagerie, le scanner permet de mettre en évidence des signes d'AVC ischémique ou hémorragique. L'ANGIO-TDM montre les sténoses et occlusions des carotides internes, et/ou des portions proximales des artères cérébrales antérieures et moyennes. Elle permet la visualisation du réseau vasculaire de suppléance : réseau Moya Moya sous forme de structures vasculaires surnuméraires serpigineuses à l'emplacement du polygone de Willis [3].

L'IRM-ARM est actuellement un examen pouvant être réalisée en 1ère intention, avec une sensibilité et spécificité de 70 à 100%. Elle permet de mettre en évidence les lésions parenchymateuses et vasculaires. Les lésions ischémiques seront visualisées sous forme d'un hyposignal T1, hypersignal T2, FLAIR et variable en diffusion selon l'ancienneté, tandis que les lésions hémorragiques auront un signal variable en T1 et T2 selon l'ancienneté, et un hyposignal T2* constant. Les vaisseaux de Moya Moya peuvent se voire sur les séquences T1 sous forme d'images punctiformes en vide de signal, serpigineuses en incidence coronale en regard des noyaux de la base. L'ARM permet l'étude du polygone de willis, des bifurcations carotidiennes, détecte les anévrysmes, mais sa résolution reste encore inférieure à celle de l'angiographie numérisée: puisqu'elle ne détecte pas les lésions micro-anévrysmales inférieures à 3 mm, elle risque de ne pas objectiver tous les petits vaisseaux de suppléance en particulier transduraux et n'apprécie pas la circulation vasculaire extra-crânienne nécessaire pour la revascularisation chirurgicale. Au stade ultime de la maladie, on retrouve des signes d'atrophie cérébrale débutant généralement aux lobes frontaux [4]. L'artériographie est un examen de référence pour le diagnostic. Elle permet en préopératoire de visualiser la vascularisation intra et extra crânienne. Le diagnostic repose sur la présence de sténose et/ou occlusion bilatérale des siphons carotidiens et /ou portions proximales des ACM et des ACA. L'opacification au temps artériel d'un réseau vasculaire de suppléance bilatéral visible autour des lésions obstructives, qui est à la base de la classification de Suzuki et Takaku, qui ont classé cette pathologie en 6 grades de gravité croissante: Grade I: sténose des portions distales des artères carotides internes; Grade II: apparition des vaisseaux de Moya Moya; Grade III: majoration du nombre de vaisseaux; Grade IV: diminution du nombre de vaisseaux; Grade V: diminution importante du nombre de vaisseaux; Grade VI: disparition des vaisseaux Moya Moya. Le doppler transcranien fournit un moyen non invasif pour suivre les changements dans l'écoulement sanguin.

Le pronostic de Moya est difficile à prévoir car l'histoire naturelle de la maladie est mal connue. L'évolution de la maladie peut être lente avec des symptômes intermittents ou être fulminante avec un déclin neurologique rapide. Les facteurs qui influent sur l'évolution sont: le degré et la rapidité des rétrécissements artériels, la capacité du patient à développer une circulation collatérale, l'âge et l'étendue des sténoses au moment du diagnostic. Une fois le diagnostic de Moya Moya est établi, une orientation rapide du patient vers un centre de référence devra être réalisée pour mettre en place un plan thérapeutique. L'indication chirurgicale doit être discutée assez rapidement, car une intervention précoce peut rapidement améliorer l'état du patient, et prévenir une récidive ischémique [5]. Les données actuelles montrent l'importance du traitement chirurgical comme méthode de référence pour la prise en charge du syndrome de Moya en particulier chez les patients avec des symptômes progressifs et récidivants. Plusieurs techniques opératoires en utilisant le plus souvent la circulation de l'artère carotide externe. Des études à long terme ont montrée de bons résultats du traitement chirurgical (risque de 4% de complication au cours du premier mois, 96% ne présentent aucun risque au cours des 5 années suivants). Le traitement chirurgical du Moya est efficace, considéré comme un traitement durable de la maladie. Une angio-TDM ou une IRM avec des séquences ARM voire une angiographie conventionnelle est souvent réalisée un an après le traitement chirurgical, puis annuellement pour le contrôle évolutif [5].

## Conclusion

Le Moya Moya est une affection vasculaire cérébrale chronique rare, idiopathique ou secondaire, constituant une cause non négligeable d'AVC pédiatrique. Le retard diagnostique est fréquent, lié à sa symptomatologie polymorphe. L'angioscanner et l'angioMR constituent actuellement l'imagerie de référence pour le diagnostic initial et la surveillance. L'artériographie reste un gold standard pour le diagnostic et le bilan lésionnel précis de cette pathologie.

## Conflits d’intérêts

Les auteurs ne déclarent aucun conflit d'intérêts.

## References

[1] Edward R Smith, Michael Scott R (2010). Moya Moya: Epidemiology, Présentation, and Diagnosis. Neurosurgery Clinics of North America..

[2] Oillic H, Henry S, Estable B, Lapostolle C, Rivoal E, Vic P (2009). Hémiparésie brutale chez un adolescent dû à un syndrome Moya Moya. Archives de Pédiatrie..

[3] Manceau E, Giroud M, Dumas R (1997). Moyamoya disease in children: A review of the clinical and radiological features and current treatment. Childs Nerv Syst..

[4] Ben Hassin L, Mekki N, Ben Hafdhallah J, Lahmar L, Louati H, Douira W, Khomsi, Bellagha I (2012). Aspect IRM du Moya Moya chez l’enfant à propos de 5 cas..

[5] Morel C, Rousselle C, Pelissou-Guyotat I, Begey-Scherrer V, Mamelle JC, Deruty R (1999). Maladie de Moya Moya: intérêt d'un diagnostic et d'un traitement chirurgical précoces: A propos de trois observations. Archives de Pédiatrie..

